# Pediatric Coronavirus Disease 2019: Why Is It Less Severe and Frequent in Children than Adults?

**DOI:** 10.34172/jrhs.2021.53

**Published:** 2021-06-01

**Authors:** Zohreh Jadali

**Affiliations:** ^1^Department of Immunology, School of Public Health, Tehran University of Medical Sciences. Tehran, Iran

## Dear Editor-in-Chief

 The new severe acute respiratory syndrome coronavirus 2 (SARS-CoV-2) was first detected among humans in China in the winter of 2019, and it was found that it could cause the disease named coronavirus disease 2019 (COVID-19). The virus has spread across the world with unprecedented speed and in the space of a few short months. It has also grown into a global pandemic that has fundamentally changed every aspect of human life.

Based * on *the epidemiological investigation results, this disease occurs in all age groups and both genders. Nonetheless, both gender and age can influence the outcome of the disease. Emerging evidence suggests that older age and male gender are at greater risk for severe illnesses^1^. Additionally, most children are asymptomatic or have mild symptoms^2^. Therefore, an understanding of the underlying mechanisms of these differences will provide an opportunity for better care, management, and prevention of the disease.

The incidence of COVID-19 among children under 18 years is below 2.2% ^3,4^; however, these figures do not reflect the actual extent of the disease. It is anticipated that the number of children with COVID-19 is far greater than thought. This can be attributed to several factors, such as differences in symptoms between children and adults, as well as milder disease course in children that results in fewer laboratory assessments and failure of consideration.

Children of all ages (from newborns to young adults) can get COVID-19. The mean age for the affected children is 7 years (SD=3.6)^5^, and the number of cases is higher for boys than girls.^6^

 The virus is mainly transmitted through droplets and household contacts; however, other transmission routes may not be excluded. This can occur, for example, during medical procedures, fetal life, or at the time of delivery.

Similar to adults, COVID-19 can cause a wide range of symptoms in children. Fever and cough are the typical signs but clinical presentation can vary from individual to individual. Most infected children suffer from minimal or *no *symptoms and do not experience severe or fatal diseases. This does not mean that the virus cannot transmit from these patients (known as silent carriers). In contrast, they may play a major role in the spread of COVID-19 in the community. The main causes of a mild form of the disease in children have not been clearly established and multiple hypotheses have been proposed in the literature.

 Some of the theories that have been developed to explain the difference between children and adults in the severity of COVID-19 are as follows:

1) Frequent children are at risk for many pathogenic viruses and bacteria. Moreover, they are immunized with attenuated and inactivated vaccines. Activation of the immune system by these agents can induce a memory-like response that is known as trained immunity^7^. This kind of immunity may be the driver of the stronger immune responses to SARS-CoV-2 in pediatric patients with COVID-19 infection ^8^.

2) SARS-CoV-2 pathogenesis deals with the interaction between the S protein of the virus and the angiotensin-converting enzyme (ACE) 2 on the surface of the host cells, and a positive correlation has been reported between ACE2 expression and SARS-CoV-2 infection. It seems that maturational and functional differences of ACE-2 between children and adults may explain the relative resistance of pediatrics to SARS-CoV-2^9^.

3) Differences in immune responses of adults and children affect the disease susceptibility and lead to the differential numbers of cases. It is well known that the immune system of children is relatively immature and gradually develops because of exposure to a wide array of pathogens. Moreover, young children are at a greater risk of infections, compared to adults. These repeated exposures result in the stimulation of innate immunity and enhancement of nonspecific antimicrobial responses. Another possible theory is related to differences in the production of inflammatory cytokines. In comparison with adults, children have a lower proinflammatory cytokine response. This feature can have a major effect on the host susceptibility since it may alleviate the uncontrolled overproduction of inflammatory cytokines, which is closely related to the development and progression of the disease. Changes in T cell homeostasis with aging are also thought to contribute to the decline in immunity and an increase in inflammation. It must be mentioned that different subsets of T cells play a major role in the polarization of the immune responses.^10^

 4) The higher prevalence of comorbidity and underlying chronic diseases in adults is a risk factor for the severity of COVID-19 illness.

In summary, there are differences in symptoms and clinical laboratory findings between the adult and children who are infected with the COVID-19. The exact *causes* of these differences are not fully understood; however, a combination of factors, such as biochemical and immunological parameters, physiological function, and anatomical structure is thought to be responsible. Proper analysis of these differences leads to accurate diagnosis, prediction of the course and outcome of the disease, as well as the improvement of treatment approaches for COVID-19.

## Conflict of Interest

 There is no conflict of interest to declare.
